# Femoral Arterial Calcification, Atherogenic Index of Plasma, and 1-Year Mortality After Hip Fracture: A Retrospective Cohort Study

**DOI:** 10.3390/jcm15187019

**Published:** 2026-09-10

**Authors:** Ahmet Yiğitbay, Erim Can Demircan, Muhammed Fatih Civan, Alev Kural, Cemal Kural, Ali Can Koluman, Nezih Ziroğlu

**Affiliations:** 1 Department of Orthopedics and Traumatology, İzmir Tepecik Training and Research Hospital, University of Health Sciences, 35170 İzmir, Turkey; ahmetyigitbay@gmail.com; 2 Department of Orthopedics and Traumatology, Bakirkoy Dr. Sadi Konuk Training and Research Hospital, University of Health Sciences, 34147 Istanbul, Turkey; 3 Department of Biochemistry, Bakirkoy Dr. Sadi Konuk Training and Research Hospital, University of Health Sciences, 34147 Istanbul, Turkey; 4 Department of Orthopaedics and Traumatology, Acibadem University Atakent Hospital, 34303 Istanbul, Turkey; 5 Vocational School of Health Services, Department of Orthopedic Prosthetics and Orthotics, Acibadem Mehmet Ali Aydinlar University, 34638 Istanbul, Turkey

**Keywords:** hip fracture, femoral arterial calcification, Atherogenic Index of Plasma, 1-year mortality, orthogeriatric care, retrospective cohort study, single-centre setting

## Abstract

**Background and Objectives:** Femoral arterial calcification (FAC) on routine hip radiographs may provide opportunistic vascular information within orthogeriatric assessment. We evaluated the associations of FAC and the Atherogenic Index of Plasma (AIP) with 1-year mortality after hip-fracture surgery. **Materials and Methods:** This retrospective single-centre cohort included 229 patients aged 65 years or older. FAC was graded from 0 to 3 on preoperative radiographs using an exploratory semiquantitative classification. AIP was calculated as log_10_(TG/HDL-C). Logistic regression and complementary survival analyses were partially adjusted for age, sex, FAC, and AIP. Incremental discrimination was assessed using nested age + sex, age + sex + FAC, and age + sex + FAC + AIP models. **Results:** Thirty-eight patients (16.6%) died within 1 year. Interobserver agreement for FAC grading was almost perfect (κ = 0.87). Each one-grade increase in FAC was associated with mortality in the partially adjusted logistic (OR = 1.44, 95% CI 1.09–1.91) and Cox models (HR = 1.36, 95% CI 1.07–1.73). AIP was not significantly associated with mortality. AUCs were 0.726 for age + sex, 0.757 for age + sex + FAC, and 0.761 for age + sex + FAC + AIP; neither incremental increase was significant. **Conclusions:** Radiographic FAC was associated with 1-year mortality but did not demonstrate significant incremental discrimination beyond age and sex. FAC may serve as an opportunistic marker within multidisciplinary orthogeriatric assessment, not as a stand-alone prognostic tool. AIP findings remain inconclusive because sampling occurred during acute admission without standardised fasting. Prospective multicentre validation with comprehensive geriatric covariates is required.

## 1. Introduction

Hip fractures are a major cause of trauma-related morbidity, functional decline, loss of independence, and mortality among older adults. Recent analyses of the Global Burden of Disease Study have confirmed that the worldwide burden of hip fracture remains substantial, increases markedly with age, and continues to grow in ageing populations [1].

Mortality and functional recovery after hip fracture are determined by complex interactions among age, comorbidity burden, frailty, cognitive and nutritional status, pre-fracture mobility, perioperative complications, and rehabilitation potential. Accordingly, contemporary hip-fracture management extends beyond surgical fixation and requires an integrated orthogeriatric pathway based on comprehensive multidimensional assessment. Such pathways involve coordinated contributions from emergency physicians, orthopaedic surgeons, geriatricians, anaesthesiologists, nurses, physiotherapists, rehabilitation specialists, dietitians, pharmacists, and social-care professionals. Orthogeriatric co-management and integrated rehabilitation models may improve care processes, including time to surgery and length of hospital stay, and may support survival and functional recovery [2,3,4,5]. Functional autonomy is particularly relevant because pre-fracture dependency and subsequent loss of activities of daily living have been associated with 12-month mortality [2]. Therefore, any imaging-derived or biochemical indicator should be interpreted alongside comprehensive geriatric, functional, nutritional, cognitive, and social assessment rather than as an isolated predictor.

At an organisational level, hip-fracture care may also be conceptualised as a clinical network connecting acute admission, perioperative management, surgery, geriatric co-management, discharge planning, rehabilitation, and community follow-up. Clinical networks facilitate collaboration across professional and institutional boundaries, support the implementation of shared evidence-based pathways, and may reduce fragmentation of care. Systematic reviews suggest that appropriately resourced clinical networks with credible leadership, effective communication, and multidisciplinary collaboration may improve care quality and selected patient outcomes and may contribute to the quadruple aim of better outcomes, improved patient experience, efficient resource use, and professional well-being [6,7]. Within such a network, opportunistically available markers such as radiographic FAC might help identify patients who warrant closer multidimensional assessment or more intensive coordination. However, such markers cannot replace comprehensive clinical evaluation or determine care pathways independently.

Atherosclerosis and vascular calcification are associated with frailty and adverse clinical outcomes in older populations. Peripheral arterial calcification is frequently observed in patients with diabetes mellitus, chronic kidney disease, and advanced age and may reflect arterial stiffness, impaired microcirculation, tissue-perfusion abnormalities, and a greater burden of cardiovascular disease [8]. Plain radiography provides a practical and widely available means of identifying radiopaque peripheral arterial calcification, although it cannot assess arterial stenosis, blood flow, plaque morphology, or haemodynamic significance. Femoral arterial calcification (FAC) visible on routine anteroposterior hip radiographs may therefore be regarded as an opportunistic marker of vascular calcification rather than a substitute for dedicated vascular imaging. Previous studies have investigated the relationship between FAC and clinical outcomes after hip fracture. Although some have suggested an association between FAC and poorer survival, others have reported weak or nonsignificant associations, particularly in specific surgical subgroups [9,10,11]. These inconsistent findings indicate that the prognostic relevance of radiographic FAC remains uncertain and may be influenced by the broader clinical, functional, and vascular characteristics of the patient.

The Atherogenic Index of Plasma (AIP), calculated as the logarithmic transformation of the triglyceride-to-high-density lipoprotein cholesterol ratio [log_10_(TG/HDL-C)], is a lipid-derived index associated with lipoprotein particle characteristics and atherogenicity. The concept was introduced by Dobiásová and colleagues, who described its relationship with small, dense low-density lipoprotein particles and atherosclerotic risk [12]. Subsequent epidemiological and clinical studies have investigated AIP in relation to cardiometabolic outcomes, including hypertension, diabetes mellitus, and vascular events [13,14]. In the perioperative setting, emerging studies of patients undergoing noncardiac surgery have suggested that AIP may reflect cardiovascular vulnerability under surgical stress [15,16]. However, lipid measurements obtained during an acute hip-fracture admission may be affected by unknown fasting status, trauma-related physiological changes, fluid administration, nutritional status, and lipid-lowering treatment. The prognostic relevance of AIP in this setting therefore remains uncertain.

Although the literature evaluating FAC in patients with hip fracture is expanding, evidence regarding the combined evaluation of FAC and AIP—particularly in geriatric hip-fracture cohorts from Türkiye—remains limited [10,11,16]. FAC and AIP were considered potentially complementary, but conceptually distinct, markers: FAC as an opportunistic radiographic indicator of vascular calcification and AIP as a biochemical indicator of atherogenic lipid status. Nevertheless, given the multifactorial nature of mortality after hip fracture, neither marker was presumed to represent an independent prognostic factor or a clinically applicable risk-stratification tool.

Accordingly, the primary objective of this study was to evaluate the association between the presence and severity of radiographic FAC and 1-year all-cause mortality in older adults undergoing surgery for hip fracture. The secondary objectives were to evaluate the association between AIP and 1-year mortality; to compare the discriminatory performance of nested age + sex, age + sex + FAC, and age + sex + FAC + AIP models; and to examine the relationship between FAC grade and mortality using complementary categorical and time-to-event analyses. These analyses were intended to be exploratory and hypothesis-generating and were not designed to establish independent prognostic value, causal relationships, or immediate clinical utility.

## 2. Materials and Methods

### 2.1. Study Design and Patient Selection

This retrospective single-centre cohort study was conducted at Bakırköy Dr. Sadi Konuk Training and Research Hospital, a tertiary trauma referral centre in Istanbul, Türkiye. The study evaluated patients admitted with hip fracture between December 2021 and January 2025. Ethical approval was obtained from the Clinical Research Ethics Committee of the same institution (Decision No. 2026-07-06; 18 March 2026), and the study was conducted in accordance with the Declaration of Helsinki.

### 2.2. Eligibility Criteria

Patients were eligible if they were aged 65 years or older, underwent surgical treatment for a hip fracture during the study period, had an admission lipid profile containing both triglyceride and high-density lipoprotein cholesterol measurements, and had an adequate preoperative pelvis or hip radiograph permitting assessment of femoral arterial calcification.

Patients were excluded if they were treated nonoperatively; had a pathological or periprosthetic fracture, multiple trauma, missing triglyceride or high-density lipoprotein cholesterol measurements, or an unavailable or technically inadequate preoperative radiograph. The exclusion criteria were applied hierarchically and were treated as mutually exclusive for reporting the patient-selection process.

### 2.3. Data Collection

Demographic, clinical, operative, and outcome data were extracted retrospectively from electronic medical records. The recorded variables included age, sex, fracture side and type, length of hospital stay, surgical procedure, anaesthesia type, American Society of Anesthesiologists (ASA) physical status, operative duration, admission and surgery dates, mortality status and date, time from admission to death, time from admission to surgery, and postoperative complications.

Detailed information on potential confounders—including hypertension, diabetes mellitus, chronic kidney disease, coronary artery disease, carotid artery disease, and atherosclerotic disease in other vascular beds—was not systematically available. Data on dementia, frailty, nutritional status, prefracture mobility, medication use, previous lower-limb vascular procedures, and cause-specific mortality were also unavailable. Consequently, these variables could not be analysed or included as covariates, and mortality was evaluated as all-cause mortality.

### 2.4. Calculation Method of the Atherogenic Index of Plasma

TG and HDL-C measurements were obtained from blood samples collected as part of routine clinical assessment within the first 24 h after hospital admission and before surgery. Because hip fracture admissions were emergent, fasting status was neither standardised nor consistently recorded. The timing of blood sampling in relation to fluid resuscitation was also unavailable.

Before calculating AIP, TG and HDL-C concentrations were converted from mg/dL to mmol/L using the following conversion factors: TG, mg/dL/88.57; and HDL-C, mg/dL/38.67. AIP was then calculated using the following formula:

$$AIP=\log_{10}\left(\frac{TG\;\left[mmol/L\right]}{HDL-C\;\left[mmol/L\right]}\right).$$

AIP calculations were independently verified by a senior biochemistry specialist and a senior orthopaedic surgeon. AIP was analysed primarily as a continuous variable. For descriptive purposes, it was also categorised as low (<0.11), intermediate (0.11–0.21), or high (>0.21) according to previously reported thresholds [12]. Information on lipid-lowering treatment was unavailable.

### 2.5. Assessment of Femoral Arterial Calcification

FAC was evaluated on standard preoperative pelvis and hip radiographs using an exploratory semiquantitative classification adapted for the present analysis from a previously proposed but unvalidated radiographic scheme [17]. The classification was used to describe the visible extent and pattern of femoral arterial calcification on plain radiographs and has not undergone formal methodological or external validation. Accordingly, FAC grade was treated as an exploratory imaging exposure rather than as an established diagnostic or prognostic classification. Plain radiography can identify radiopaque arterial calcification but cannot directly assess luminal stenosis, plaque morphology, blood flow, or hemodynamic significance. Accordingly, radiographic FAC was treated as an opportunistic marker of vascular calcification rather than as a diagnostic measure of femoral atherosclerosis. The grading system was not independently validated against dedicated vascular imaging in the present cohort.

FAC severity was classified as follows:

- Grade 0: no visible calcific deposits;
- Grade 1: small, scattered calcific deposits;
- Grade 2: calcification involving <50% of the visible arterial wall;
- Grade 3: calcification involving ≥50% of the visible arterial wall or showing a continuous “lead-pipe” appearance.

In accordance with the source classification, patients with a visible vascular stent were assigned to Grade 3. However, because the presence of a vascular stent is not biologically equivalent to extensive arterial calcification and stent status was not stored as a separate variable in the analytical dataset, a stent-specific sensitivity analysis could not be performed. Information on previous common femoral endarterectomy, patch angioplasty, or other lower-limb vascular procedures was similarly unavailable.

Radiographs were independently assessed by two senior orthopaedic surgeons who were blinded to clinical outcomes and to each other’s assessments. Disagreements were resolved by consensus. Interobserver agreement before consensus was quantified using Cohen’s kappa coefficient. FAC grade was entered as an ordinal variable coded from 0 to 3 in the primary analysis, providing an effect estimate for each one-grade increase. In a sensitivity analysis, FAC grade was modelled categorically, with Grade 0 as the reference category.

### 2.6. Outcome Assessment

The primary outcome was 1-year all-cause mortality, defined as death occurring within 365 days after hospital admission. Mortality status and dates of death were obtained from official civil-registry records available through the national population registration system. The national mortality registry was last queried on 20 March 2026.

Because the latest eligible admission occurred in January 2025, all 229 patients had at least 365 days of potential follow-up at the time of the registry query. Therefore, all 229 patients were included in the fixed 1-year mortality analyses. A total of 45 deaths were recorded during the entire available observation period. Of these, 38 occurred within 365 days after admission and constituted primary-endpoint events; seven occurred after 365 days and contributed only to the descriptive overall-mortality count.

For the Kaplan–Meier and Cox regression analyses, follow-up was administratively censored at 365 days. In-hospital mortality, 30-day mortality, and overall mortality during the available observation period were evaluated as secondary descriptive outcomes. Cause-specific mortality was unavailable.

### 2.7. Surgical Procedures

Surgical procedures were performed using standard institutional techniques by different surgical teams. Patients undergoing proximal femoral nail or dynamic hip screw fixation were operated on in the supine position using a traction table, whereas patients undergoing hemiarthroplasty or total hip arthroplasty were operated on in the lateral decubitus position. The anaesthesia technique was selected by the attending anesthesiologist according to routine clinical assessment. The clinical considerations underlying anaesthesia selection were not systematically available for analysis.

### 2.8. Statistical Analysis

Statistical analyses were performed using Python 3.12 with SciPy 1.18.0, statsmodels 0.14.6, and scikit-learn 1.9.0. Mortality ascertainment was complete for all 229 patients; therefore, the full cohort was used for baseline summaries, fixed 1-year endpoint comparisons, regression analyses, ROC analysis, calibration assessment, internal validation, and time-to-event analyses.

Continuous variables were summarised using mean ± standard deviation and median with interquartile range or range, as appropriate. Categorical variables were presented as numbers and percentages. Continuous variables were compared using the Mann–Whitney U test, whereas categorical variables were compared using the chi-square test or Fisher’s exact test, as appropriate. Mean age was compared between patients with and without femoral arterial calcification (FAC) using Welch’s *t* test. All statistical tests were two-sided, and *p* < 0.05 was considered statistically significant.

The primary analysis evaluated the association between FAC grade and 1-year all-cause mortality using binary logistic regression, with death within 365 days after hospital admission defined as the primary outcome. Age, sex, FAC grade, and the Atherogenic Index of Plasma (AIP) were selected a priori on clinical grounds and entered simultaneously into the model. Results were reported as odds ratios (ORs) with 95% confidence intervals (CIs). FAC grade was modelled as an ordinal variable coded from 0 to 3 in the primary analysis, providing an OR per one-grade increase. In a sensitivity analysis, FAC grade was modelled categorically, with Grade 0 used as the reference category.

Because data on major potential confounders—including cardiovascular disease, diabetes mellitus, renal dysfunction, dementia, frailty, nutritional status, prefracture mobility, and medication use—were unavailable, the regression estimates were interpreted as partially adjusted associations rather than independent prognostic effects. Secondary analyses evaluated the association of AIP with 1-year mortality, the incremental discriminatory performance of the prespecified nested models, the categorical FAC estimates, and the association between FAC grade and survival in complementary time-to-event analyses.

Discrimination for 1-year mortality was evaluated for AIP alone, FAC alone, FAC + AIP, and the partially adjusted age + sex + FAC + AIP model using receiver operating characteristic (ROC) curves and areas under the curve (AUCs). AUC 95% CIs were estimated using 2000 bootstrap resamples, and pairwise differences between correlated AUCs were evaluated using DeLong’s test. Predicted-probability thresholds were selected using the Youden index and were considered exploratory.

Model calibration was evaluated using the calibration intercept and slope, Brier score, and Hosmer–Lemeshow goodness-of-fit test. Internal validation of the partially adjusted model was performed using 2000 bootstrap resamples to estimate optimism-corrected AUC, Brier score, and calibration slope.

For the complementary time-to-event analyses, follow-up was administratively censored at 365 days. Kaplan–Meier curves and the log-rank test were used to compare 1-year survival across FAC grades. A partially adjusted Cox proportional-hazards model containing age, sex, FAC grade, and AIP was used to estimate hazard ratios (HRs) with 95% CIs.

## 3. Results

### 3.1. Patient Selection and Cohort Characteristics

**Figure 1:**
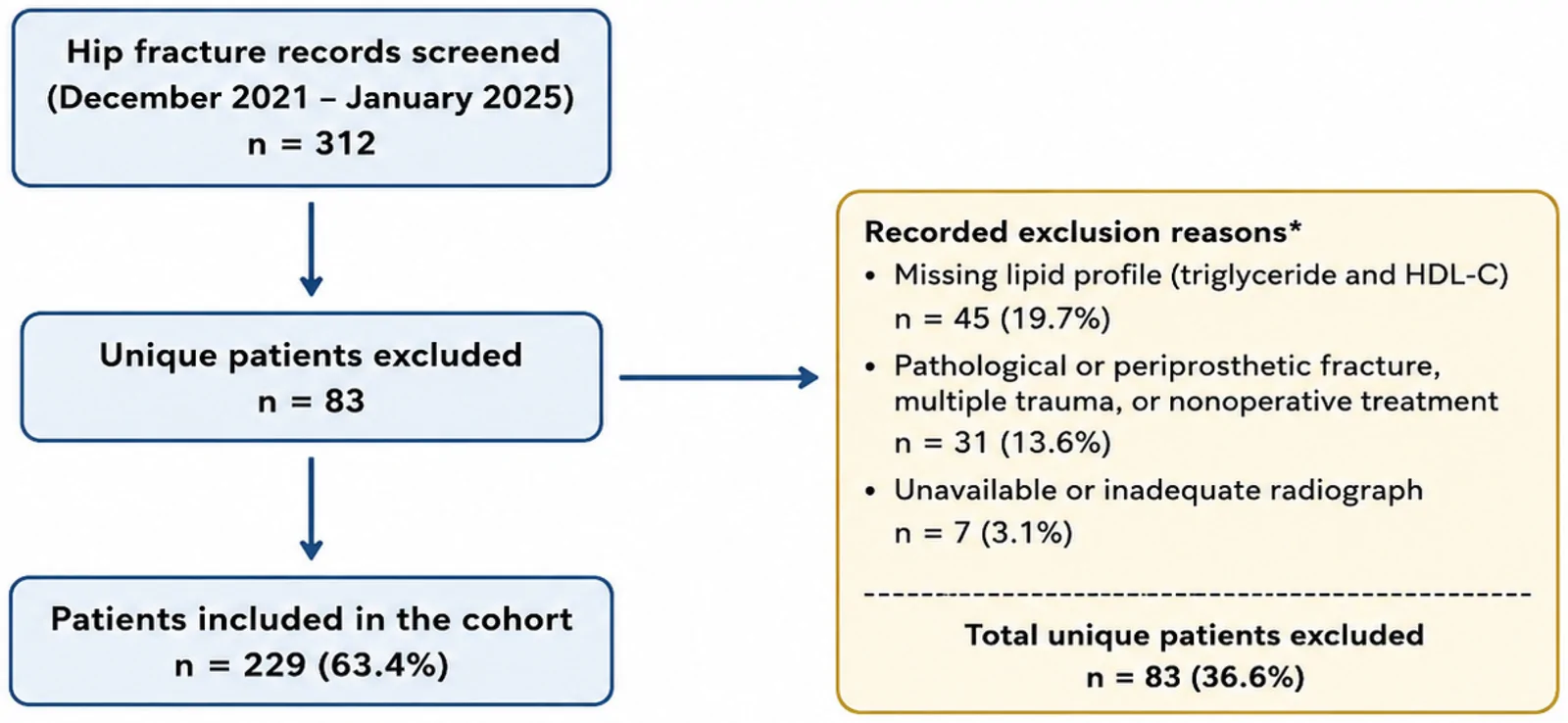
Patient-selection flow diagram. * Exclusion categories were applied hierarchically and were mutually exclusive.

A total of 312 patient records were screened. Of these, 229 patients met all eligibility criteria and were included in the analysis. Eighty-three patients were excluded because of missing lipid profiles (*n* = 45); pathological or periprosthetic fractures, multiple trauma, or nonoperative treatment (*n* = 31); or unavailable or technically inadequate radiographs (*n* = 7). The exclusion categories were applied hierarchically and were mutually exclusive (Figure 1).

A total of 229 patients were included in the analysis. The cohort comprised 152 women (66.4%) and 77 men (33.6%). The mean age was 80.8 ± 7.8 years, with a median of 81 years (interquartile range [IQR], 75–87; range, 65–97). There were 128 right-sided fractures (55.9%) and 101 left-sided fractures (44.1%). Intertrochanteric fractures were present in 142 patients (62.0%), femoral neck fractures in 80 (34.9%), and subtrochanteric fractures in 7 (3.1%) (Table 1).

### 3.2. Mortality and Perioperative Outcomes

Thirty-eight patients died within 365 days after admission, corresponding to a 1-year all-cause mortality rate of 16.6% (38/229). Eight patients (3.5%) died within 30 days, all during the index hospitalisation. Overall, 45 deaths (19.7%) were recorded during the available observation period; seven occurred more than 365 days after admission and were not counted as primary-endpoint events.

Regional, combined, or other anaesthesia was used in 206 patients (90.0%), whereas 23 patients (10.0%) received general anaesthesia (Table 2).

### 3.3. Femoral Arterial Calcification

Interobserver agreement for FAC grading before consensus was almost perfect (Cohen’s κ = 0.87). FAC was absent (Grade 0) in 132 patients (57.6%) and present (Grades 1–3) in 97 (42.4%). Patients with FAC were older than those without FAC (82.1 ± 7.7 vs. 79.8 ± 7.8 years; *p* = 0.032). Sex distribution (*p* = 0.338) and fracture laterality (*p* = 0.068) did not differ significantly. FAC grade was associated with 1-year mortality (χ^2^ = 10.07, df = 3; *p* = 0.018), with mortality increasing across grades (Table 3).

### 3.4. Atherogenic Index of Plasma

Mean AIP was 0.065 ± 0.275, and median AIP was 0.052 (IQR, −0.110 to 0.213; range, −0.708 to 0.877). Mean AIP was 0.072 ± 0.234 among patients who died within 1 year and 0.064 ± 0.282 among those who survived beyond 1 year; the distributions did not differ (Mann–Whitney U *p* = 0.530). AIP risk category was not associated with mortality (χ^2^ = 3.12, df = 2; *p* = 0.210) (Table 4).

### 3.5. Logistic Regression Analysis

**Figure 2:**
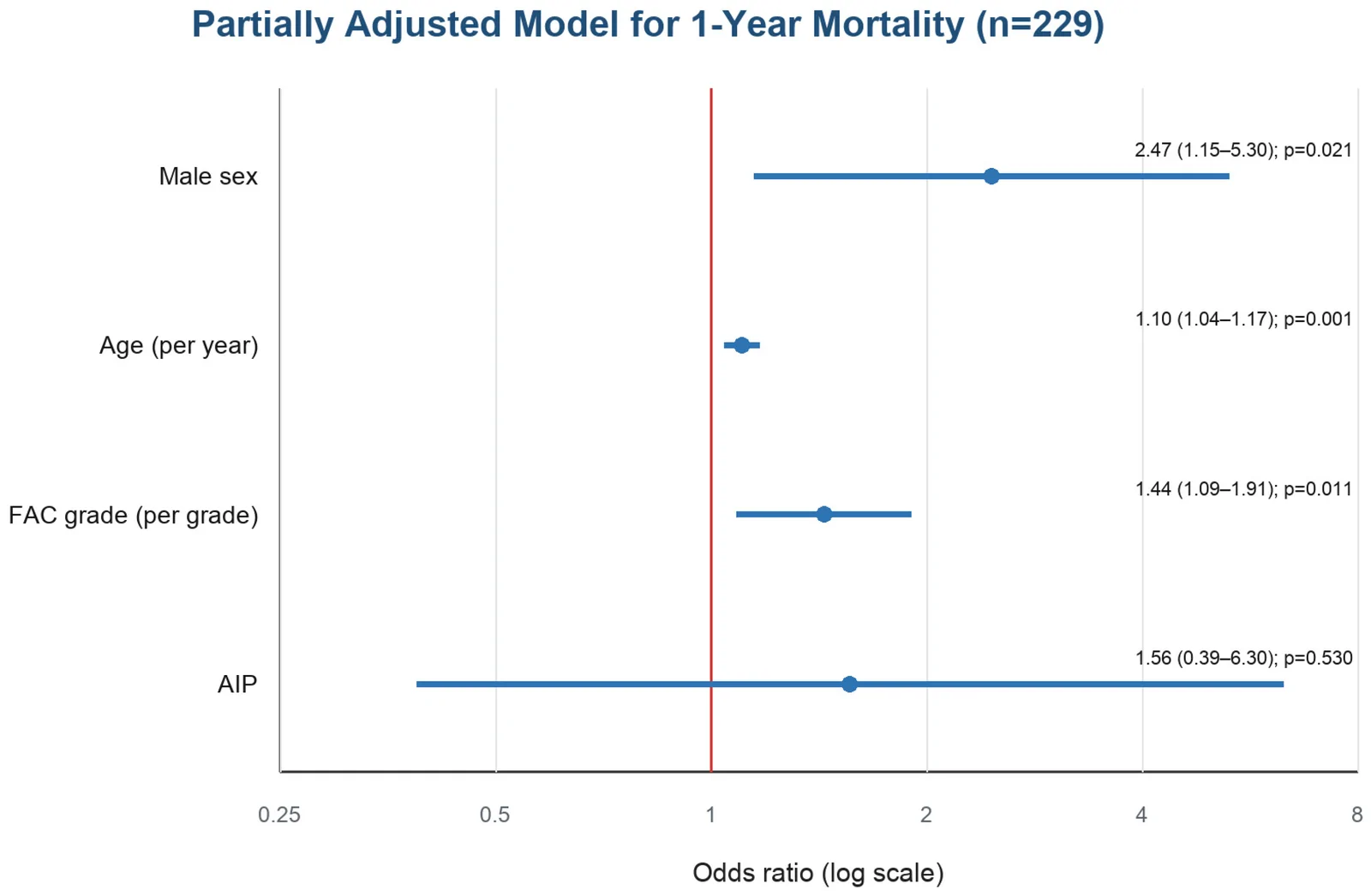
Forest plot of the partially adjusted logistic regression model for 1-year all-cause mortality (*n* = 229). Points indicate odds ratios, and horizontal lines indicate 95% confidence intervals. The vertical reference line indicates an odds ratio of 1. The model included age, sex, FAC grade, and AIP simultaneously. Female sex was the reference category. Abbreviations: AIP, Atherogenic Index of Plasma; CI, confidence interval; FAC, femoral arterial calcification; OR, odds ratio.

In the partially adjusted logistic regression model, each one-grade increase in FAC was associated with higher odds of 1-year mortality (OR = 1.44, 95% CI 1.09–1.91; *p* = 0.011). Age (OR = 1.10 per year, 95% CI 1.04–1.17; *p* < 0.001) and male sex (OR = 2.47, 95% CI 1.15–5.30; *p* = 0.021) were also associated with mortality, whereas AIP was not (OR = 1.56, 95% CI 0.39–6.30; *p* = 0.530) (Table 5; Figure 2).

### 3.6. Discrimination, Calibration, and Internal Validation

**Figure 3:**
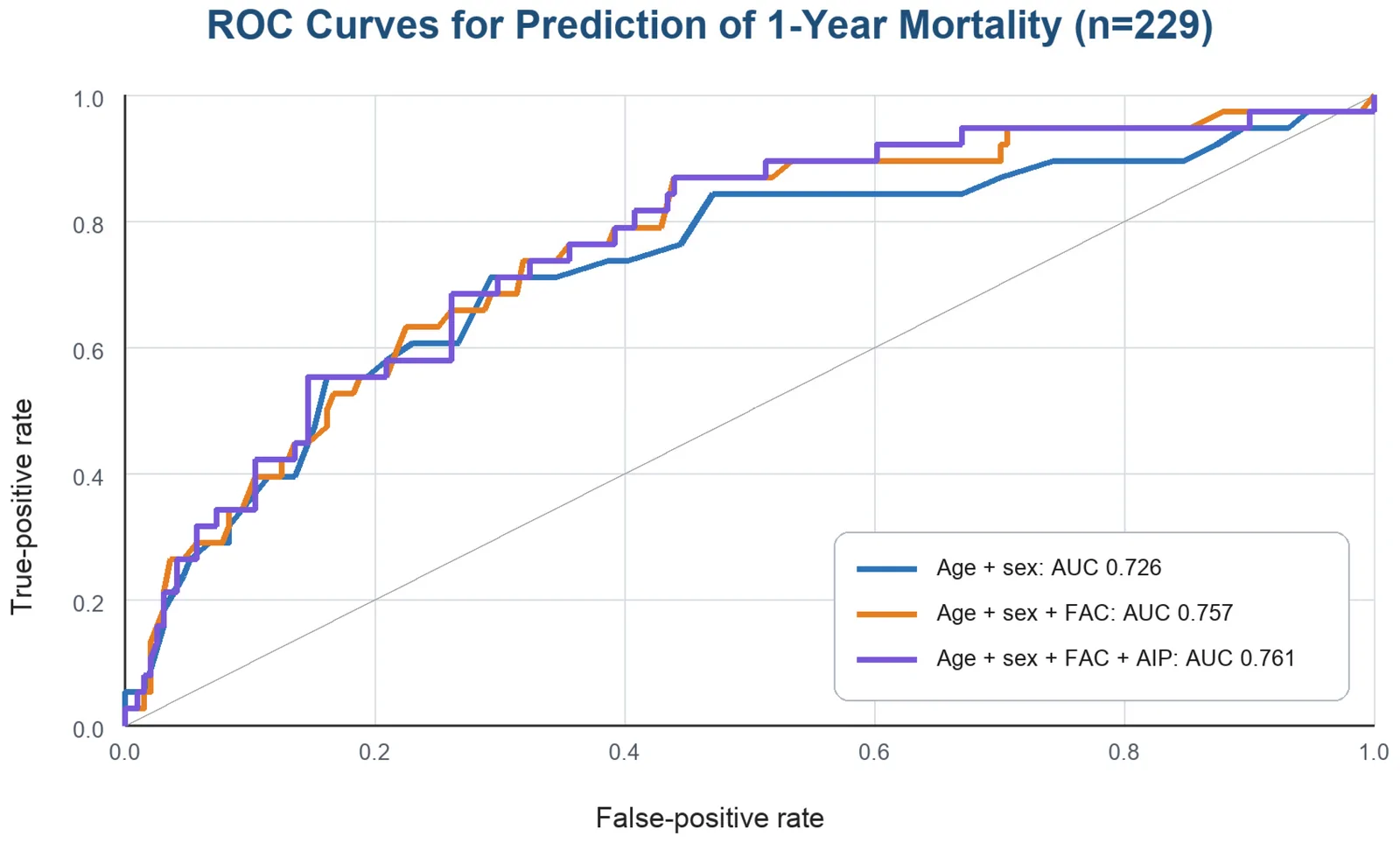
Receiver operating characteristic curves for three nested models predicting 1-year all-cause mortality: age + sex (AUC = 0.726), age + sex + FAC (AUC = 0.757), and age + sex + FAC + AIP (AUC = 0.761) (*n* = 229). Adding FAC to age and sex did not significantly improve discrimination (DeLong *p* = 0.233), and adding AIP to the age + sex + FAC model provided no significant further improvement (DeLong *p* = 0.389). Abbreviations: AIP, Atherogenic Index of Plasma; AUC, area under the curve; FAC, femoral arterial calcification; ROC, receiver operating characteristic.

AIP alone showed limited discrimination (AUC = 0.532, bootstrap 95% CI 0.437–0.620). FAC alone had an AUC of 0.642 (95% CI 0.544–0.731), and FAC + AIP had an AUC of 0.661 (95% CI 0.564–0.757). Adding AIP to FAC did not significantly improve discrimination (DeLong *p* = 0.322). The age + sex + FAC + AIP model had an apparent AUC of 0.761 (95% CI 0.671–0.843), exceeding FAC alone (*p* = 0.014) and FAC + AIP (*p* = 0.040) (Figure 3).

The apparent Brier score was 0.120, and the Hosmer–Lemeshow test did not indicate lack of fit (χ^2^ = 8.03; *p* = 0.431). After 2000 bootstrap resamples, the optimism-corrected AUC was 0.735, the corrected Brier score was 0.128, and the corrected calibration slope was 0.876. The exploratory FAC + AIP probability threshold of 0.225 yielded 52.6% sensitivity and 76.4% specificity.

The age + sex model had an AUC of 0.726 (bootstrap 95% CI 0.625–0.814). Adding FAC increased the AUC to 0.757 (95% CI 0.665–0.835), although this improvement was not statistically significant (DeLong *p* = 0.233). The addition of AIP to the age + sex + FAC model resulted in an AUC of 0.761 (95% CI 0.672–0.841), with no significant incremental improvement in discrimination (DeLong *p* = 0.389).

### 3.7. Time-to-Event Analysis

**Figure 4:**
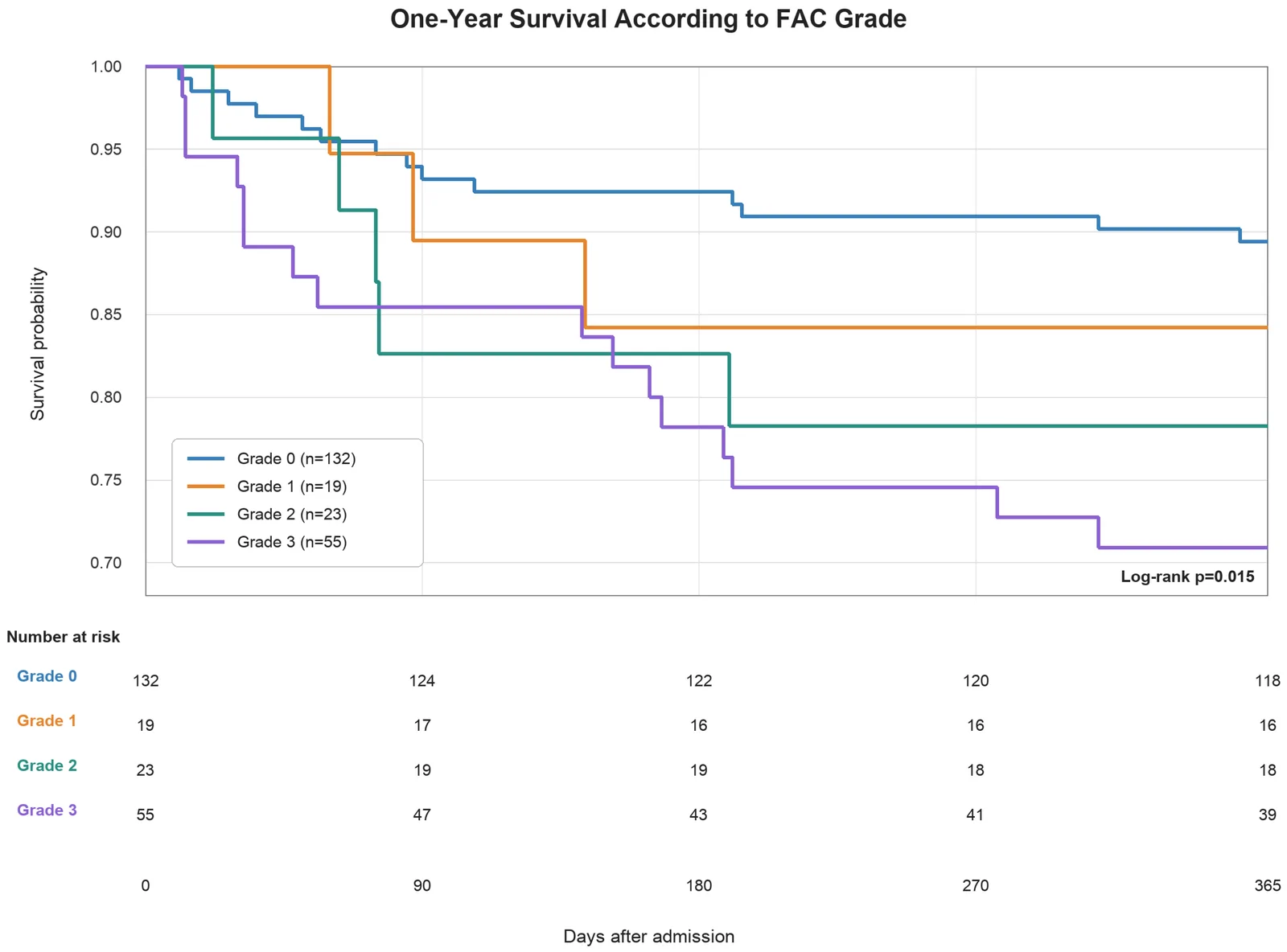
Kaplan–Meier survival curves according to FAC grade, with follow-up administratively censored at 365 days. The number-at-risk table is presented below the survival curves. Survival distributions differed across FAC grades (log-rank *p* = 0.015). Abbreviation: FAC, femoral arterial calcification.

Kaplan–Meier survival differed across FAC grades during the 365-day follow-up period (log-rank χ^2^ = 10.47, df = 3; *p* = 0.015) (Figure 4). In the complementary Cox model adjusted for age, sex, and AIP, each one-grade increase in FAC was associated with a higher hazard of 1-year mortality (HR = 1.36, 95% CI 1.07–1.73; *p* = 0.013). Age (HR = 1.09 per year, 95% CI 1.04–1.15; *p* < 0.001) and male sex (HR = 2.07, 95% CI 1.09–3.95; *p* = 0.027) were also associated with mortality, whereas AIP was not (HR = 1.50, 95% CI 0.46–4.93; *p* = 0.499).

## 4. Discussion

### 4.1. Principal Findings

In this retrospective cohort, increasing radiographic FAC grade was associated with higher 1-year all-cause mortality in partially adjusted logistic and Cox regression analyses. However, adding FAC to age and sex increased the AUC only from 0.726 to 0.757, and this incremental improvement was not statistically significant. Adding AIP produced a minimal further increase in the AUC to 0.761. No statistically significant association between AIP and mortality was detected. These findings suggest that FAC may reflect cumulative vascular and systemic vulnerability but do not establish FAC as an independent prognostic factor or demonstrate significant incremental discrimination beyond age and sex. The findings should therefore be regarded as exploratory and hypothesis-generating.

### 4.2. Comparison with Existing Literature

Older adults with hip fracture constitute a high-risk population characterised by frailty, functional impairment, and substantial comorbidity. Mortality and recovery are influenced by interacting demographic, clinical, nutritional, functional, and perioperative factors [18,19]. Graif et al. found that concomitant upper-extremity fractures prolonged hospitalisation but were not independently associated with mortality after adjustment for age, sex, fracture type, and comorbidity burden [20]. Khalil et al. reported 30-day and 1-year mortality rates of 3.5% and 14.1%, respectively, after hip arthroplasty for femoral neck fracture and identified postoperative medical complications as an important determinant of mortality [21]. Their 1-year estimate was broadly comparable with the 16.6% observed in the present cohort. Other studies have demonstrated associations of mortality with nutritional and immunological status [22], respiratory and cardiac complications, readmission, dementia, age, sex, albumin, and haemoglobin [23], as well as ASA classification, heart failure, chronic pulmonary disease, and body mass index [24]. Functional ambulation, premorbid living situation, weight-bearing restrictions, delirium, and heart failure may also influence rehabilitation requirements [25], while cognitive, depressive, and nutritional findings have been associated with 1-year mortality [26]. Collectively, this evidence supports interpreting additional biomarkers or imaging findings within a comprehensive clinical and orthogeriatric assessment rather than in isolation.

AIP has been investigated as a marker of atherogenic dyslipidaemia and cardiovascular risk [27,28], and emerging evidence suggests a possible association with adverse outcomes, including myocardial injury after noncardiac surgery [15]. In a large peritoneal-dialysis cohort, Lan et al. found that higher baseline AIP was associated with all-cause mortality, cardiovascular mortality, and peritonitis after adjustment for cardiovascular disease, diabetes, laboratory variables, and lipid-lowering treatment [29]. In the present acute hip-fracture cohort, no statistically significant association between AIP and 1-year mortality was detected, and adding AIP to the age + sex + FAC model produced only a minimal, non-significant increase in discrimination. Differences from previous findings may reflect variation in study populations, underlying renal and metabolic characteristics, sampling conditions, and covariate adjustment.

AIP principally reflects chronic atherogenic lipid status, whereas mortality after hip fracture may arise from multiple competing cardiovascular and non-cardiovascular mechanisms. In this study, lipid measurements were obtained during acute admission without standardised fasting and may have been affected by trauma-related inflammatory and physiological responses, fluid resuscitation, nutritional status, and ongoing lipid-lowering treatment. The absence of a statistically significant association should therefore be interpreted cautiously and should not be regarded as evidence that AIP has no prognostic value under standardised measurement conditions or in other populations.

Radiographic FAC, in contrast, represents visible arterial mineralisation and may reflect cumulative vascular and systemic disease burden associated with cardiovascular and all-cause mortality [30]. It can be identified opportunistically on routine hip radiographs without additional imaging or radiation exposure. The FAC classification used in this study was adapted for exploratory analysis from a previously proposed radiographic scheme and has not undergone formal external validation. Although interobserver agreement was almost perfect, reproducibility between observers does not establish diagnostic validity, prognostic validity, or equivalence to dedicated vascular imaging. Furthermore, plain radiography cannot assess luminal stenosis, plaque morphology, non-calcified plaque, blood-flow impairment, or haemodynamic significance. Undocumented previous vascular interventions or stents may also have resulted in exposure misclassification. FAC should therefore be interpreted as an exploratory radiographic marker of visible vascular calcification rather than as a diagnostic measure of femoral atherosclerosis.

Previous studies evaluating FAC after hip fracture have produced inconsistent results, ranging from significant associations to weak or non-significant findings [9,10,11,31]. These differences may reflect variation in patient characteristics, calcification definitions and grading methods, event rates, previous vascular procedures, follow-up duration, and covariate adjustment. In the present study, the association between increasing FAC grade and 1-year mortality was directionally consistent across logistic regression, Kaplan–Meier, and Cox analyses. Nevertheless, adding FAC to age and sex increased the AUC from 0.726 to 0.757 without a statistically significant incremental improvement. This finding suggests that FAC may capture aspects of cumulative vascular and systemic vulnerability but does not establish that it provides prognostic discrimination beyond basic demographic variables.

Interpretation of the observed association remains constrained by the limited number of deaths and the absence of detailed information on comorbidity, frailty, nutritional and cognitive status, functional autonomy, medication use, and previous vascular procedures. FAC may partly represent the combined burden of age, vascular disease, frailty, and comorbidity rather than an independent prognostic effect. The estimates should therefore be regarded as partially adjusted and hypothesis-generating, pending confirmation in larger prospective studies with comprehensive orthogeriatric assessment and validated vascular imaging.

The combined assessment of AIP and FAC was based on a biologically plausible but exploratory multimarker concept integrating biochemical and structural features of atherosclerotic burden. Previous studies have suggested that cardiovascular risk assessment may benefit from combining biochemical markers with imaging-derived measures [28,32]. In the nested-model analysis, adding FAC to age and sex increased the AUC from 0.726 to 0.757; however, this incremental improvement was not statistically significant (DeLong *p* = 0.233). Adding AIP produced only a minimal further increase in the AUC to 0.761, which was also not statistically significant (DeLong *p* = 0.389). These findings indicate that the apparent discrimination of the full model was driven largely by age and sex and do not establish that FAC provides significant incremental discrimination beyond these basic demographic variables. Bootstrap internal validation also demonstrated some optimism in the apparent performance of the full model. Nevertheless, these findings do not establish the general superiority of one marker over the other or demonstrate that AIP has no prognostic value. Neither FAC nor AIP, individually or in combination, should be used as a stand-alone predictor or interpreted using the exploratory predicted-probability threshold without external validation.

Established clinical variables such as ASA physical status and time to surgery are commonly considered in perioperative risk assessment [33,34]. Nevertheless, the predictive value of individual clinical variables may be limited when considered in isolation, particularly among frail older patients with multiple interacting comorbidities. Recent evidence supports a broader approach integrating clinical, functional, nutritional, biochemical, and imaging information when evaluating outcomes after hip fracture [20,21,22,23,24,25,26,31,32,35].

In the nested-model analysis, adding FAC to age and sex increased the AUC from 0.726 to 0.757; however, this incremental improvement was not statistically significant (DeLong *p* = 0.233). Adding AIP produced only a minimal further increase in the AUC to 0.761, which was also not statistically significant (DeLong *p* = 0.389). These findings indicate that the apparent discrimination of the full model was driven largely by age and sex and do not establish that FAC provides significant incremental discrimination beyond these basic demographic variables. Nevertheless, the absence of a statistically significant association for AIP should not be interpreted as evidence that AIP has no prognostic value, particularly given the acute clinical setting and non-standardised fasting conditions.

### 4.3. Perspectives for Clinical Practice

Hip-fracture care requires coordinated management across emergency assessment, perioperative optimisation, surgery, geriatric co-management, nursing care, rehabilitation, nutritional support, discharge planning, and community follow-up. Within such a multidimensional pathway, radiographic FAC may be available without additional imaging, cost, or radiation exposure and could serve as an opportunistic signal of underlying vascular and systemic vulnerability. Its presence, particularly at higher grades, might prompt clinicians to ensure that cardiovascular comorbidity, renal function, frailty, nutritional status, cognition, medication burden, and functional reserve have been comprehensively assessed.

However, the present findings do not support using FAC to determine treatment, surgical timing, level of care, discharge destination, or rehabilitation intensity independently. FAC did not provide statistically significant incremental discrimination beyond age and sex, and relevant clinical and geriatric confounders were unavailable. It should therefore be interpreted only as contextual information within comprehensive geriatric assessment rather than as a screening test or stand-alone risk score.

From an organisational perspective, the incorporation of opportunistically available information into structured orthogeriatric pathways may support communication among orthopaedic surgeons, geriatricians, anaesthesiologists, radiologists, nurses, rehabilitation professionals, and primary-care teams. Any future use of FAC would require a standardised assessment method, clear reporting procedures, prospective validation, and demonstration that communicating the finding produces clinically meaningful changes in care processes or patient outcomes. Similar caution applies to AIP, whose value should be reassessed under standardised sampling conditions before consideration within clinical pathways.

### 4.4. Strengths and Limitations

The strengths of this study include complete ascertainment of 1-year mortality for all included patients, blinded FAC assessment by two observers with almost-perfect interobserver agreement, consistent findings across logistic and time-to-event analyses, and evaluation of incremental discrimination using nested models. Nevertheless, several limitations affect the interpretation and generalisability of the findings.

The retrospective, single-centre design precludes causal inference and may have introduced selection bias. Although lipid testing was routinely requested as part of the admission laboratory assessment, complete triglyceride and HDL-C results were not retrievable for all screened patients. Restriction of the analysis to patients with a complete lipid profile and an adequate radiograph may therefore have selected a subgroup that differed systematically from excluded patients. Conducting the study at a large tertiary trauma referral centre serving a heterogeneous population does not eliminate the limitations associated with local clinical practices, referral patterns, and a single-centre cohort. Consequently, the findings may not be directly generalisable to institutions with different patient populations or orthogeriatric pathways.

Comprehensive and consistently recorded information was unavailable for several major determinants of mortality after hip fracture, including cardiovascular and renal disease, diabetes mellitus, frailty, nutritional and cognitive status, functional autonomy, pre-fracture mobility, medication use, and previous vascular procedures. These factors could not be incorporated reliably into the multivariable models, leaving substantial potential for residual confounding. FAC may therefore reflect the cumulative burden of age, vascular disease, frailty, and comorbidity rather than an independent prognostic effect. In addition, only 38 deaths occurred during the prespecified 1-year follow-up period. This limited the statistical power and precision of the regression estimates, particularly in the categorical FAC analysis, in which several confidence intervals were wide. The categorical estimates should therefore be considered exploratory. Because cause-specific mortality data were unavailable, cardiovascular and non-cardiovascular deaths could not be distinguished.

FAC was assessed on routine plain radiographs using an adapted semiquantitative classification that was not externally validated against dedicated vascular imaging. Although interobserver agreement was high, reproducibility does not establish diagnostic or prognostic validity. Plain radiography cannot characterise luminal stenosis, blood flow, plaque morphology, non-calcified plaque, or haemodynamic significance. Previous vascular interventions and vascular stents were not systematically documented and may have resulted in exposure misclassification. The FAC classification should therefore be regarded as an exploratory radiographic measure of visible vascular calcification rather than a validated diagnostic measure of femoral atherosclerosis.

AIP was calculated from lipid measurements obtained during acute hip-fracture admission without standardised fasting conditions. Triglyceride and HDL-C concentrations may have been influenced by acute trauma, inflammatory and physiological responses, fluid resuscitation, nutritional status, and lipid-lowering treatment. These factors may have reduced the reliability of AIP as an indicator of usual atherogenic lipid status. Accordingly, the absence of a statistically significant association in this cohort should not be interpreted as evidence that AIP has no prognostic value under standardised measurement conditions or in other populations.

FAC provided only modest discrimination, and adding FAC to age and sex did not result in a statistically significant increase in AUC. Adding AIP to the age + sex + FAC model produced only a minimal and non-significant further increase. Internal validation also indicated optimism in the apparent performance of the full model, and no external validation was performed. Neither FAC nor the combined model should therefore be used as a stand-alone clinical risk-stratification or decision-making tool. These hypothesis-generating findings require confirmation in larger, adequately powered, prospective multicentre studies incorporating comprehensive geriatric assessment, standardised lipid sampling, validated vascular imaging methods, adjudicated causes of death, and external model validation.

## 5. Conclusions

In this retrospective single-centre cohort of older adults undergoing hip-fracture surgery, increasing radiographic FAC grade was associated with higher 1-year all-cause mortality in partially adjusted analyses. However, FAC did not provide statistically significant incremental discrimination beyond age and sex, and no statistically significant association between AIP and mortality was detected. These findings do not establish FAC as an independent prognostic factor or AIP as lacking prognostic value. Radiographic FAC may be considered an opportunistically available indicator of vascular and systemic vulnerability, but it should be interpreted only within comprehensive multidimensional and multiprofessional orthogeriatric assessment and not used as a stand-alone risk-stratification or decision-making tool. Larger prospective multicentre studies incorporating validated FAC assessment, standardised lipid sampling, comprehensive geriatric covariates, and external validation are required before either marker can be incorporated into clinical care pathways.

## Figures and Tables

**Table 1:** Baseline demographic and clinical characteristics.

Variable	Value
Number of patients	229
Age, mean ± SD	80.8 ± 7.8
Age, median (IQR; range)	81 (75–87; 65–97)
Female sex, *n* (%)	152 (66.4%)
Male sex, *n* (%)	77 (33.6%)
Right-sided fracture, *n* (%)	128 (55.9%)
Left-sided fracture, *n* (%)	101 (44.1%)
Intertrochanteric fracture, *n* (%)	142 (62.0%)
Femoral neck fracture, *n* (%)	80 (34.9%)
Subtrochanteric fracture, *n* (%)	7 (3.1%)
Length of stay, mean ± SD	13.1 ± 8.5 days
Length of stay, median (range)	11 (1–60) days

Data are presented as number (%), mean ± standard deviation, or median (interquartile range or range), as appropriate. Abbreviations: IQR, interquartile range; SD, standard deviation.

**Table 2:** Perioperative variables and clinical outcomes.

Variable	Value
General anaesthesia, *n* (%)	23 (10.0%)
Regional/combined/other anaesthesia, *n* (%)	206 (90.0%)
1-year mortality, *n* (%)	38 (16.6%)
30-day mortality, *n* (%)	8 (3.5%)
Overall mortality, *n* (%)	45 (19.7%)
Any postoperative complication, *n* (%)	71 (31.0%)
ICU admission, *n* (%)	44 (19.2%)
Postoperative infection, *n* (%)	24 (10.5%)
Surgical procedure, *n* (%)	
Proximal femoral nailing (PFN)	141 (61.5%)
Hemiarthroplasty	68 (29.7%)
Dynamic hip screw (DHS)	9 (3.9%)
Total hip arthroplasty (THA)	7 (3.1%)
Cannulated screw fixation	4 (1.7%)
Operative duration, mean ± SD	102.7 ± 37.4 min
Operative duration, median (range)	100 (35–310) min

Data are presented as number (%) or mean ± standard deviation, unless otherwise indicated. Abbreviations: DHS, dynamic hip screw; ICU, intensive care unit; PFN, proximal femoral nailing; SD, standard deviation; THA, total hip arthroplasty.

**Table 3:** FAC grade distribution and 1-year mortality.

FAC Grade	Patients, *n*	1-Year Deaths, *n*	Mortality
Grade 0	132	14	10.6%
Grade 1	19	3	15.8%
Grade 2	23	5	21.7%
Grade 3	55	16	29.1%
Total	229	38	16.6%

Mortality percentages were calculated within each FAC grade. Abbreviations: FAC, femoral arterial calcification.

**Table 4:** AIP distribution and mortality association.

Variable	Value
Mean ± SD	0.065 ± 0.275
Median (IQR)	0.052 (−0.110 to 0.213)
Range	−0.708 to 0.877
Low risk, *n* (%)	127 (55.5%)
Intermediate risk, *n* (%)	43 (18.8%)
High risk, *n* (%)	59 (25.8%)
AIP in 1-year deaths	0.072 ± 0.234
AIP in 1-year survivors	0.064 ± 0.282
Mann–Whitney U test	*p* = 0.530
Risk category vs. mortality	χ^2^ = 3.12; *p* = 0.210

AIP was calculated as log_10_(TG/HDL-C), with lipid concentrations expressed in mmol/L. Continuous AIP distributions were compared using the Mann–Whitney U test, and AIP risk categories were compared using the chi-square test. Abbreviations: AIP, Atherogenic Index of Plasma; HDL-C, high-density lipoprotein cholesterol; IQR, interquartile range; SD, standard deviation; TG, triglyceride.

**Table 5:** Partially adjusted logistic regression model for 1-year mortality.

Variable	OR	95% CI	*p*-Value
AIP	1.56	0.39–6.30	0.530
FAC grade, per one-grade increase	1.44	1.09–1.91	0.011
Age, per year	1.10	1.04–1.17	<0.001
Male sex	2.47	1.15–5.30	0.021

With Grade 0 as the reference, adjusted ORs were 1.69 (95% CI 0.40–7.13; *p* = 0.474) for Grade 1, 1.60 (95% CI 0.48–5.28; *p* = 0.441) for Grade 2, and 3.14 (95% CI 1.33–7.38; *p* = 0.009) for Grade 3. The model included age, sex, FAC grade, and AIP simultaneously. FAC was entered as an ordinal variable coded from 0 to 3; the reported OR represents each one-grade increase. Female sex was the reference category. Abbreviations: AIP, Atherogenic Index of Plasma; CI, confidence interval; FAC, femoral arterial calcification; OR, odds ratio.

## Data Availability

The data supporting the findings of this study are not publicly available because they contain sensitive patient information and are subject to institutional and ethical restrictions. De-identified data may be made available by the corresponding author upon reasonable request and subject to approval by the relevant institutional and ethics authorities.

## Abbreviations

The following abbreviations are used in this manuscript:

AIP	Atherogenic Index of Plasma
ASA	American Society of Anesthesiologists
AUC	Area Under the Curve
CI	Confidence Interval
DHS	Dynamic Hip Screw
FAC	Femoral Arterial Calcification
HDL-C	High-Density Lipoprotein Cholesterol
HR	Hazard Ratio
ICU	Intensive Care Unit
LDL	Low-Density Lipoprotein
OR	Odds Ratio
PFN	Proximal Femoral Nailing
ROC	Receiver Operating Characteristic
SD	Standard Deviation
TG	Triglyceride
THA	Total Hip Arthroplasty
